# Clinical outcomes in cancer patients with COVID‐19

**DOI:** 10.1002/cnr2.1413

**Published:** 2021-08-19

**Authors:** Amelia Sawyers, Margaret Chou, Paul Johannet, Nicholas Gulati, Yingzhi Qian, Judy Zhong, Iman Osman

**Affiliations:** ^1^ Ronald O. Perelman Department of Dermatology NYU Grossman School of Medicine New York USA; ^2^ Department of Medicine NYU Grossman School of Medicine New York USA; ^3^ Department of Population Health NYU Grossman School of Medicine New York USA

**Keywords:** active malignancy, cancer, coronavirus disease 2019, outcomes, survivor

## Abstract

**Background:**

Early reports on cancer patients with coronavirus disease 2019 (COVID‐19) corroborated speculation that cancer patients are at increased risk for becoming infected with severe acute respiratory syndrome coronavirus 2 (SARS‐CoV‐2) and developing severe COVID‐19. However, cancer patients are a heterogeneous population and their corresponding risk may be different.

**Aim:**

To compare COVID‐19 presentation in patients with active malignancy to those with a history of cancer to determine the impact of cancer status on COVID‐19 outcomes in the two groups.

**Methods and results:**

Of the 6724 patients who were hospitalized at NYU Langone Health (3/16/20‐7/31/20) and tested positive for SARS‐CoV‐2, 580 had either active cancer (*n* = 221) or a history of cancer (*n* = 359). We compared the baseline clinicodemographic characteristics and hospital courses of the two groups. We studied the relationship between cancer status and the rate of admission to the intensive care unit (ICU), use of invasive mechanical ventilation (IMV), and all‐cause mortality. The two groups had similar laboratory results associated with COVID‐19 infection, incidence of venous thromboembolism, and incidence of severe COVID‐19. Active cancer status was not associated with the rate of ICU admission (*p* = .307) or use of IMV (*p* = .236), but was significantly associated with worse all‐cause mortality in both univariate and multivariate analysis with odds ratios of 1.48 (95% confidence interval [CI]: 1.04–2.09; *p* = .028) and 1.71 (95% CI: 1.12–2.63; *p* = .014), respectively.

**Conclusion:**

Active cancer patients had worse survival outcomes compared to patients with a history of cancer despite similar COVID‐19 disease characteristics in the two groups. Our data suggest that cancer care should continue with minimal interruptions during the pandemic to bring about response and remission as soon as possible.

## 1 INTRODUCTION

As of March 2021, more than 123 000 000 people worldwide have become infected with severe acute respiratory syndrome coronavirus 2 (SARS‐CoV‐2). The resultant coronavirus disease 2019 (COVID‐19) has led to over 2 700 000 deaths.^1^ Cancer patients are vulnerable to infection with community acquired respiratory viruses due to a variety of factors including immunocompromised status secondary to malignancy itself, predisposition for malnutrition, and immunosuppressive treatments.^2^, ^3^ These same factors confer an increased risk for developing complications of respiratory viral infections, such as lower respiratory tract illness and hypoxemic respiratory failure, and raised concern that cancer patients would be particularly susceptible to severe COVID‐19.^4^, ^5^

Earlier reports from China suggested that both patients with active cancer or a history of malignancy are at heightened risk for contracting SARS‐CoV‐2 and suffering worse disease outcomes.^6^, ^7^, ^8^, ^9^, ^10^ These findings were subsequently corroborated by studies from around the world.^11^, ^12^, ^13^, ^14^, ^15^ However, more recent data revealed similar morbidity and mortality of hospitalized COVID‐19 patients with active malignancy compared to individuals without cancer matched by age and number of comorbidities.^16^ This discrepancy raises a broader question of whether cancers and their treatment regimens should be considered as risk factors for COVID‐19 on an individual basis or in aggregate.

Cancer patients are a heterogeneous population and their corresponding risk for severe COVID‐19 is likely different. The extant data do not provide a complete picture of the clinical characteristics and disease trajectories of COVID‐19 in patients with active malignancy compared to those with a history of cancer. Until vaccination is widespread, and since cancer patients might be at significantly elevated risk for poor COVID‐19 outcomes, it is imperative to determine whether the same risk applies to patients with a history of malignancy. This could inform clinical decision‐making about the frequency of health care visits and timing of surveillance imaging for cancer survivors, who constitute a large population of at least 16 900 000 individuals in the United States of America.^17^

A report on all COVID‐19 patients at NYU Langone Health between March 1 and April 8, 2020 documented that age and comorbidities were associated with worse outcomes and found that 24.3% of hospitalized patients died.^18^ In this study, we aimed to evaluate whether and to what degree cancer status is associated with known variables of worse COVID‐19 outcomes in a cohort of hospitalized patients.

## 2 METHODS

### 2.1 Study population

We included adult patients who were hospitalized between March 16, 2020 and July 31, 2020 at NYU Langone Hospital ‐ Tisch/Kimmel, NYU Langone Orthopedic Hospital, NYU Langone Hospital ‐ Brooklyn, and NYU Langone Hospital ‐ Long Island. To be included in this study, patients needed to have a current or past diagnosis of cancer, and a laboratory confirmed diagnosis of SARS‐CoV‐2 infection as determined by reverse transcription polymerase chain reaction (RT‐PCR) of a nasopharyngeal swab specimen. Cancer status was categorized as active cancer versus a history of cancer based on the available data, which was sometimes limited because many individuals received their oncology care at other institutions. Patients were classified as having active malignancy if (1) they received treatment within 6 months of their COVID‐19 diagnosis but did not achieve cure, (2) they had measurable disease, or (3) the outpatient or inpatient notes documented that disease was present at the time of their hospitalization. Patients were classified as having a history of cancer if (1) there was no evidence of measurable disease, (2) there were no treatments administered within 6 months of their COVID‐19 diagnosis, and (3) the outpatient or inpatient notes documented that disease was in remission at the time of hospitalization. Patients with non‐invasive tumors and those with non‐metastatic, non‐melanoma skin cancers were excluded from this study.

### 2.2 Data collection

We extracted all patient‐level data through manual review of the electronic medical record used at NYU Langone Health (Epic Systems, Verona, WI). Baseline clinical and demographic characteristics included age, sex, ethnicity, body mass index (BMI), and cancer type, which was classified as solid versus hematologic. We retrieved information on preexisting medical comorbidities known to correspond with worse outcomes in COVID‐19. These included atherosclerotic disease, congestive heart failure (CHF), chronic kidney disease (CKD), chronic obstructive pulmonary disease (COPD) or asthma, cerebrovascular accident (CVA), diabetes, hypertension, immunocompromised status, liver disease, and venous thromboembolism (VTE). Additionally, we examined nine different serum laboratory tests that are frequently abnormal in patients with COVID‐19: D‐dimer, C‐reactive protein (CRP), lactate dehydrogenase (LDH), alanine aminotransferase (ALT), aspartate aminotransferase (AST), ferritin, interleukin 6 (IL‐6), white blood cell (WBC) count, and absolute lymphocyte count. We obtained code status orders from the time of admission and discharge or death, which were recorded as full code (CPR and intubation if required), do not resuscitate (DNR), or do not intubate (DNI). Finally, we gathered information about inpatient investigational treatments for COVID‐19. Pertinent definitions are presented in Appendix S1.

### 2.3 Clinical outcomes

We evaluated the relationship between cancer status and admission to the intensive care unit (ICU), length of ICU stay, use of invasive mechanical ventilation (IMV), occurrence of VTE, length of hospitalization, COVID‐19 severity, and all‐cause mortality. VTE was defined as an image‐based detection of pulmonary embolism (PE) or deep vein thrombosis (DVT), or the initiation of therapeutic anticoagulation while hospitalized, since clinical suspicion was often substituted for imaging studies during the surge in hospitalizations in New York City (NYC). Using previously published criteria from NYU Langone Health, patients were classified with severe COVID‐19 if they had dyspnea (RR > 30/min), hypoxia (<93% O2 saturation), >50% lung involved in imaging in 24–48 h, respiratory failure, septic shock or multiorgan dysfunction.^19^

### 2.4 Statistical analyses

We present categorical variables as frequencies with proportions and continuous variables as median values with standard deviations. We used the Chi‐square test (or the Fisher exact test when appropriate) to compare categorical data, and Student's *t* test for continuous data. We used multivariable Cox proportional hazards models to analyze the association between cancer status and all‐cause mortality, ICU admission, and use of IMV. The multivariable models were adjusted for baseline clinical and demographic characteristics and pre‐existing medical comorbidities. The full list of covariates is provided in Appendix S1. Patients who had a code status of DNR/DNI were excluded from the multivariable models evaluating ICU admission and use of IMV. Every patient was included in the multivariable model examining all‐cause mortality regardless of code status. Missing laboratory values were excluded. Statistical tests were two‐sided and *p* < .05 was considered significant. All analyses were performed using R version 4.0.2.

## 3 RESULTS

**FIGURE 1:**
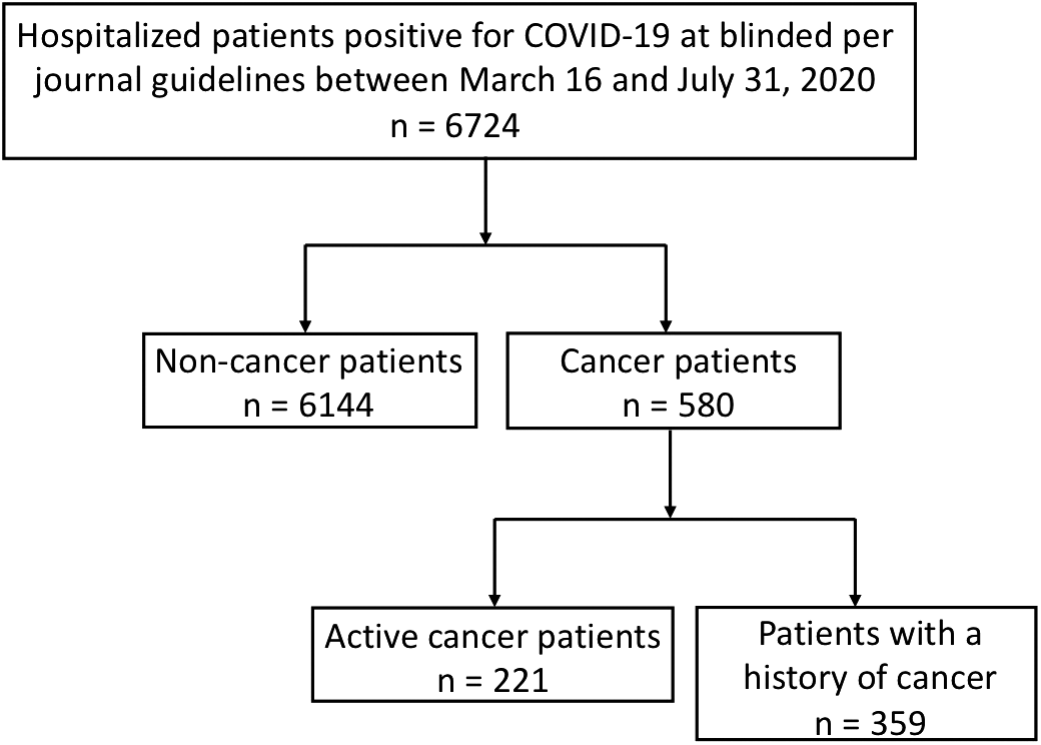
Cohort selection

6724 patients with PCR‐proven COVID‐19 were hospitalized at NYU Langone Health during the study period. 221 (3.3%) had active malignancy and 359 (5.3%) had a history of cancer (Figure 1 and Table 1). 178 (80.5%) active cancer patients were hospitalized for COVID‐19 and 43 (19.5%) were hospitalized for other reasons, but had a positive RT‐PCR and were either asymptomatic or had mild symptoms of COVID‐19. In individuals with a history of cancer, 305 (85.0%) were admitted to the hospital for COVID‐19 and 54 (15.0%) were COVID‐19 positive but admitted for a different condition. All patients who tested positive for COVID‐19, regardless of reason for admission, were included in morbidity and mortality analysis.

**TABLE 1:** Baseline characteristics of 580 COVID‐19 cancer patients

	Active (*n* = 221)	History of cancer (*n* = 359)	*p*‐value
Median age [IQR]	71.0 [62.0–79.0]	77.0 [67.0–84.0]	<.001
Male sex	126 (57.0)	198 (55.2)	.725
Ethnicity			.356
Non‐Hispanic white	114 (53.3)	199 (58.2)	
Hispanic	46 (21.5)	66 (19.3)	
Non‐Hispanic Black	33 (15.4)	56 (16.4)	
Non‐Hispanic Asian or Pacific Islander	20 (9.3)	21 (6.1)	
Non‐Hispanic American Indian/Alaska Native	1 (0.5)	0 (0.0)	
Cancer type			<.001
Solid	153 (69.2)	333 (92.8)	
Hematologic	68 (30.8)	26 (7.2)	
Current or former smoker	100 (46.5)	150 (42.6)	.412
Median body mass index (BMI) [IQR]	25.73 [22.2–29.7]	26.55 [23.4–31.5]	.009
Comorbidities			
Atherosclerotic disease	67 (30.3)	157 (43.7)	.002
Congestive heart failure (CHF)	25 (11.3)	63 (17.5)	.056
Chronic kidney disease (CKD)	47 (21.3)	108 (30.1)	.026
Chronic obstructive pulmonary disease (COPD) or asthma	51 (23.1)	89 (24.8)	.712
Cerebrovascular accident (CVA)	28 (12.7)	33 (9.2)	.235
Diabetes	75 (33.9)	147 (40.9)	.110
Hypertension	144 (65.2)	283 (78.8)	<.001
Immunocompromised	45 (20.4)	39 (10.9)	.002
Liver disease	29 (13.1)	22 (6.1)	.006
Venous thromboembolic event (VTE)	40 (18.1)	46 (12.8)	.105
Code status			.004
Full code	149 (67.4)	285 (79.4)	
Baseline DNR/DNI	9 (4.1)	12 (3.3)	
Changed to DNR/DNI during hospitalization	63 (28.5)	62 (17.3)	
Median follow‐up time, alive only [IQR]	94.00 [35.5–120.5]	83.00 [19.0–116.0]	.086

### 3.1 Patients with a history of cancer have risk factors associated with worse outcomes

In our study cohort, patients with history of cancer have risk factors associated with worse outcomes. Patients with a history of cancer were significantly older than those with active malignancy (77.0 vs. 71.0 years; *p* < .001), and more often had a preexisting diagnosis of hypertension (78.8% vs. 65.2%; *p* = <.001), atherosclerotic disease (43.7% vs. 30.3%, *p* = .002), or chronic kidney disease (30.1% vs. 21.3%; *p* = .026) (Table 1).

### 3.2 COVID‐19 related morbidity was similar in patients with active cancer and those with a history of malignancy

Active cancer patients and those with a history of cancer had similar hospital courses. Laboratory results were comparable between the two groups (Table S1). Median peak CRP was 151.1 in active cancer patients and 164.0 in those with a history of cancer (*p* = .633). The median peak ferritin was also similar (1268.4 vs. 1101.0; *p* = .295). The median absolute lymphocyte count nadir was equivalent between groups (0.6 vs. 0.6; *p* = 0.783), and the median peak D‐dimer was nearly equivalent (1462.0 vs. 1349.0; *p* = .667).

There was no significant difference in ICU admission (18.1% vs. 22.0%; *p* = .305) or developing severe COVID‐19 (59.3% vs. 65.2%; *p* = .18) between patients with active malignancies and those with a history of cancer (Table 2). The two groups received similar pharmacologic treatments and total number of medications for COVID‐19 (*p* = .961). They also had an equivalent median length of hospital stay (7.0 vs. 7.0 days; *p* = .594). However, patients with a history of cancer were more likely to be full code (79.4% vs. 67.4%; *p* = .002) and have a longer ICU stay (10.5 vs. 6.0 days; *p* = .028). There was a trend toward significance where patients with a history of cancer were more likely to be intubated (13.6% vs. 20.1%; *p* = .06). However, in an analysis of only full code patients, cancer status was not associated with the incidence of ICU admission in univariable or multivariable analyses (*p* = .520 and .307, respectively) or use of IMV in univariable or multivariable analyses (*p* = .232 and *p* = .236, respectively).

**TABLE 2:** Hospital course and management decisions

	Active (*n* = 221)	History of cancer (*n* = 359)	*p*‐value
Mortality	90 (40.7)	114 (31.8)	.035
Severe COVID‐19	131 (59.3)	234 (65.2)	.18
ICU admission	40 (18.1)	79 (22.0)	.305
Invasive respiratory intervention	30 (13.6)	72 (20.1)	.060
Median length of stay [IQR]	7.0 [3.0–13.0]	7.0 [3.0–13.0]	.594
Median ICU length of stay [IQR]	6.00 [2.8–13.3]	10.50 [4.0–20.8]	.028
Thromboembolic event	51 (23.1)	81 (22.6)	.944
Pre‐hospital therapeutic anticoagulation	45 (20.4)	64 (17.8)	.516
Number of COVID‐19 treatments received [IQR]	2 [2–3]	2 [2–3]	.961
COVID‐19 treatments received			
Antibiotics	129 (58.4)	229 (63.8)	.224
Antivirals	15 (6.8)	28 (7.8)	.773
Corticosteroids	25 (11.3)	45 (12.5)	.758
Convalescent plasma	12 (5.4)	18 (5.0)	.979
Monoclonal antibodies	18 (8.1)	38 (10.6)	.411

### 3.3 Patients with active malignancy had higher all‐cause mortality than individuals with a history of cancer

A total of 204 (35.2%) patients died during the study period (90 active cancer patients and 114 patients with a history of cancer). And 183 patients died from complications of COVID‐19 and 13 from complications related to their cancer. Of these 13 patients, 7 were admitted for COVID‐19 and 6 for cancer‐related reasons, despite having mild‐to‐severe COVID‐19 symptoms. Compared to individuals with a history of cancer, a significantly higher percentage of active cancer patients died in the total cohort (40.7% vs. 31.8%; *p* = .035) and in the subset of patients with severe COVID‐19 (59.5% vs. 47.8%; *p* = .042) (Table 2). Active cancer status was significantly associated with worse mortality in both univariate and multivariate analysis with ORs of 1.48 (95% CI: 1.04–2.09; *p* = .028) and 1.71 (95% CI: 1.12–2.63; *p* = .014), respectively (Table 3).

**TABLE 3:** Multivariate analysis of primary outcomes for active cancer patients compared to those of patients with a history of cancer

Outcomes	Univariable odds ratio (95% CI)	*p*‐value	Multivariable adjusted odds ratio (95% CI)	*p*‐value
All‐cause mortality	1.48 (1.04–2.09)	.028	1.71 (1.12–2.63)	.014
ICU admission	0.86 (0.54–1.35)	.520	0.75 (0.43–1.3)	.307
Mechanical ventilation	0.75 (0.46–1.20)	.232	0.70 (0.39–1.25)	.236

Older age was independently associated with an increased risk of all‐cause mortality in all patients (*p* = .01 in active cancer patients; *p* = .007 in patients with a history of malignancy) (Tables 4 and 5). In those with a history of malignancy, male sex, history of diabetes mellitus, and an immunocompromising comorbidity were also associated with an increased risk of mortality (*p* = .003, *p* = .001, and *p* = .018, respectively) (Tables 4 and 5).

**TABLE 4:** Multivariable baseline predictors of mortality in active cancer patients

	Odds ratio [95% CI]	*p*‐value
Age	1.04 (1.01–1.07)	.010
Male sex	1.40 (0.74–2.64)	.300
Current or former smoker	1.14 (0.60–2.18)	.682
Diabetes	1.09 (0.54–2.17)	.810
COPD or asthma	1.94 (0.95–4.00)	.070
Hypertension	0.84 (0.41–1.70)	.627
Chronic heart failure	1.38 (0.50–3.80)	.530
Atherosclerotic disease	1.55 (0.76‐3.20)	.230
Venous thromboembolic event	0.95 (0.43–2.04)	.891
Cerebrovascular accident	1.28 (0.52–3.19)	.591
Chronic kidney disease	0.70 (0.32–1.48)	.358
Liver disease	1.04 (0.41–2.58)	.932
Immunocompromised	2.10 (0.97–4.63)	.062
BMI	1.00 (0.95–1.06)	.992
Hematologic malignancy	1.31 (0.66–2.60)	.438

**TABLE 5:** Multivariable baseline predictors of mortality in patients with a history of cancer

	Odds ratio [95% CI]	*p*‐value
Age	1.04 (1.01–1.06)	.007
Male sex	2.22 (1.31–3.83)	.003
Current or former smoker	0.83 (0.49–1.40)	.484
Diabetes	2.51 (1.47–4.35)	.001
COPD or asthma	1.31 (0.72–2.38)	.373
Hypertension	2.02 (0.96–4.44)	.071
Chronic heart failure	1.84 (0.90–3.75)	.093
Atherosclerotic disease	0.77 (0.44–1.34)	.352
Venous thromboembolic event	1.27 (0.59–2.67)	.532
Cerebrovascular accident	1.36 (0.60–3.04)	.451
Chronic kidney disease	0.58 (0.32–1.04)	.074
Liver disease	0.08 (0.01–0.40)	.006
Immunocompromised	2.96 (1.21–7.46)	.018
BMI	0.94 (0.90–0.99)	.026
Hematologic malignancy	0.99 (0.35–2.64)	.983

At presentation, 559 (96.4%) patients were full code; only 9 (4.1%) active cancer patients and 12 (3.3%) patients with a history of cancer were DNR/DNI (*p* = .820) (Table 1). During the course of hospitalization, significantly more active cancer patients changed their code status to DNR/DNI (28.5% vs. 17.3%; *p* = .002). Across the entire cohort, the incidence of death was significantly higher among patients whose code status changed to DNR/DNI compared to those who were full code (65.6% vs. 25.1%; *p* < .001). However, there was no significant difference in mortality between active cancer patients and patients with a history of cancer whose code status changed to DNR/DNI (68.3% vs. 62.9%; *p* = .659) or who remained full code (27.5% vs. 23.9%; *p* = .473).

## 4 DISCUSSION

We report that cancer patients had similar manifestations of COVID‐19 regardless of their cancer status as evidenced by their serum laboratory values, incidence of severe COVID‐19, total number and types of treatments received, and rates of ICU admission. Despite the minimal differences in morbidity between groups, active cancer status was significantly associated with higher all‐cause mortality. This has important implications for cancer management during the pandemic as it demonstrates the necessity of minimizing interruptions to essential oncologic services that can lead to response and remission.

Active cancer patients suffered higher all‐cause mortality even though patients with a history of cancer were significantly older and more often carried a preexisting diagnosis of hypertension, atherosclerotic disease, or chronic kidney disease, all of which are known risk factors for poor prognosis in COVID‐19.^20^ One potential explanation is that patients with active malignancy were more likely to have an immune compromising condition in addition to cancer. Data suggest that immunosuppressed populations are at increased risk for persistent infection and poor outcomes.^21^, ^22^, ^23^ Specifically, one third of active cancer patients in our cohort had hematologic malignancies, which are themselves associated with immune suppression. Prior studies have shown that individuals with hematologic cancers experience poor COVID‐19 outcomes, which could be due to an inability to clear virus.^24^, ^25^

Advance care planning is often an expected part of a cancer diagnosis, and it is also relevant to COVID‐19 patients because they can decompensate quickly and require higher levels of care. However, not all patients want these measures, so it is important to examine COVID‐19 outcomes in the context of patients' goals of care. We found that patients who converted to DNR/DNI had significantly higher mortality than individuals who were full code. Importantly, in this subset of patients, there was no difference in mortality between active cancer patients and patients with a history of cancer. Any patient in this DNR/DNI group, regardless of cancer status, was at equally high risk of death. However, active cancer patients were significantly more likely to convert to DNR/DNI. Therefore, the larger proportion of active cancer patients compared to patients with a history of cancer who converted to DNR/DNI, and the associated increase in mortality with this conversion may account for the higher all‐cause mortality that we observed in the active cancer group. It is unclear why active cancer patients were more likely to change their code status. One explanation is that they were less able to withstand severe COVID‐19 so escalation of care was deemed futile. Another possibility is that they elected for less aggressive interventions in the setting of end‐stage malignancy, which naturally increases awareness about advance care planning. Regardless, the fact that only 4.1% of patients with active cancer had a DNR/DNI order on admission while 28.5% converted to DNR/DNI during the hospitalization highlights the need to improve proactive advance care planning during the COVID‐19 pandemic.

Both active cancer patients and patients with a history of cancer had worse survival than the general population hospitalized with COVID‐19 in NYC around the same time.^18^, ^26^ A report on all COVID‐19 patients at our institution found that 24.3% of hospitalized patients died, which contrasts to our rates of 40.7% and 31.8% for active cancer patients and patients with a history of cancer, respectively.^18^ Our findings support the growing body of evidence that malignancy portends worse COVID‐19 prognosis, and demonstrate that the risk may even apply to those without active disease.

Survival in our cohort was worse than that of other hospitalized cancer patients from the same time period. Most studies reported an in‐hospital mortality ranging between 20% and 30%.^6^, ^9^, ^11^, ^12^, ^16^ Several factors might have contributed to this discrepancy. First, the median age of our patients was higher than it was in multiple other studies.^6^, ^9^, ^11^ Second, the majority of our patients had multiple preexisting conditions.^27^ A poor medical baseline might have contributed to our patients' worse survival. Importantly, although mortality was higher in our cohort, this was not a consequence of resource availability as our institution faced challenges during the surge in NYC, but not to the point of suffering significant supply shortages or needing to ration care.^18^

There are several limitations in our study. Many patients in our cohort received their oncology care outside of NYU, which limited available information. We limited our dataset to patients with a positive RT‐PCR test, which has a well‐described false negative rate.^14^ In addition, many of our patients were hospitalized before glucocorticoids became the standard of care for treating hypoxic respiratory failure from SARS‐CoV‐2.^28^ The available evidence suggests that systemic steroids confer a survival benefit in critically ill COVID‐19 patients.^29^ More data are also needed to understand the short‐ and long‐term implications for cancer patients who receive corticosteroids but are also undergoing anti‐cancer treatments potentially compromised by steroid use.^30^

In conclusion, this is, to our knowledge, the first study to directly compare the clinical characteristics and illness phenotype of COVID‐19 in patients with active malignancy versus a history of cancer. Our data suggest that the morbidity does not differ based on cancer status, but mortality is worse in individuals with active cancer. This speaks to the importance of continuing cancer care during the pandemic with as few interruptions as possible. Since both groups appear to be at higher risk for poor COVID‐19 outcomes relative to the general population, the need to proceed with routine management and monitoring should be complemented by aggressive measures to reduce nosocomial exposures and continue to expedite vaccination of active cancer patients and patients with a history of cancer. These measures remain particularly important because of the emergence of new viral variants that decrease vaccine efficacy, unequal vaccine rollout around the world, uncertainty of asymptomatic infection or transmission post vaccination and new data on the long‐term impacts of COVID‐19.

## CONFLICT OF INTEREST

The authors have stated explicitly that there are no conflicts of interest in connection with this article.

## AUTHOR CONTRIBUTIONS

All authors had full access to the data in the study and take responsibility for the integrity of the data and the accuracy of the data analysis. *Conceptualization*, A.S., M.C., P.J., I.O.; *Investigation*, A.S., M.C., P.J, N.G., Y.Q., J.Z., I.O.; *Formal Analysis*, Y.Q., J.Z.; *Writing ‐ Original Draft*, A.S., M.C., P.J, N.G., Y.Q., J.Z., I.O.; *Writing ‐ Review & Editing*, A.S., M.C., P.J, N.G., Y.Q., J.Z., I.O.; *Supervision*, I.O.

## ETHICAL STATEMENT

The NYU Langone Health institutional review board approved this study (IRB #10362). Informed written consent was obtained from all patients.

## Supporting information


**Table S1.** Laboratory values across hospitalization in COVID‐19 cancer patients (*n* = 580).

**Appendix S1.** Supporting information.

## Data Availability

The data from this study can be made available upon reasonable request to the corresponding author. The data are not publicly available due to privacy or ethical restrictions.
