# Supplementary figures and images for: Clinical outcomes in cancer patients with COVID‐19
Source: Cancer Rep (Hoboken). 2021 Aug 19;4(6):e1413. doi: 10.1002/cnr2.1413 (PMC8420395; doi:10.1002/cnr2.1413)

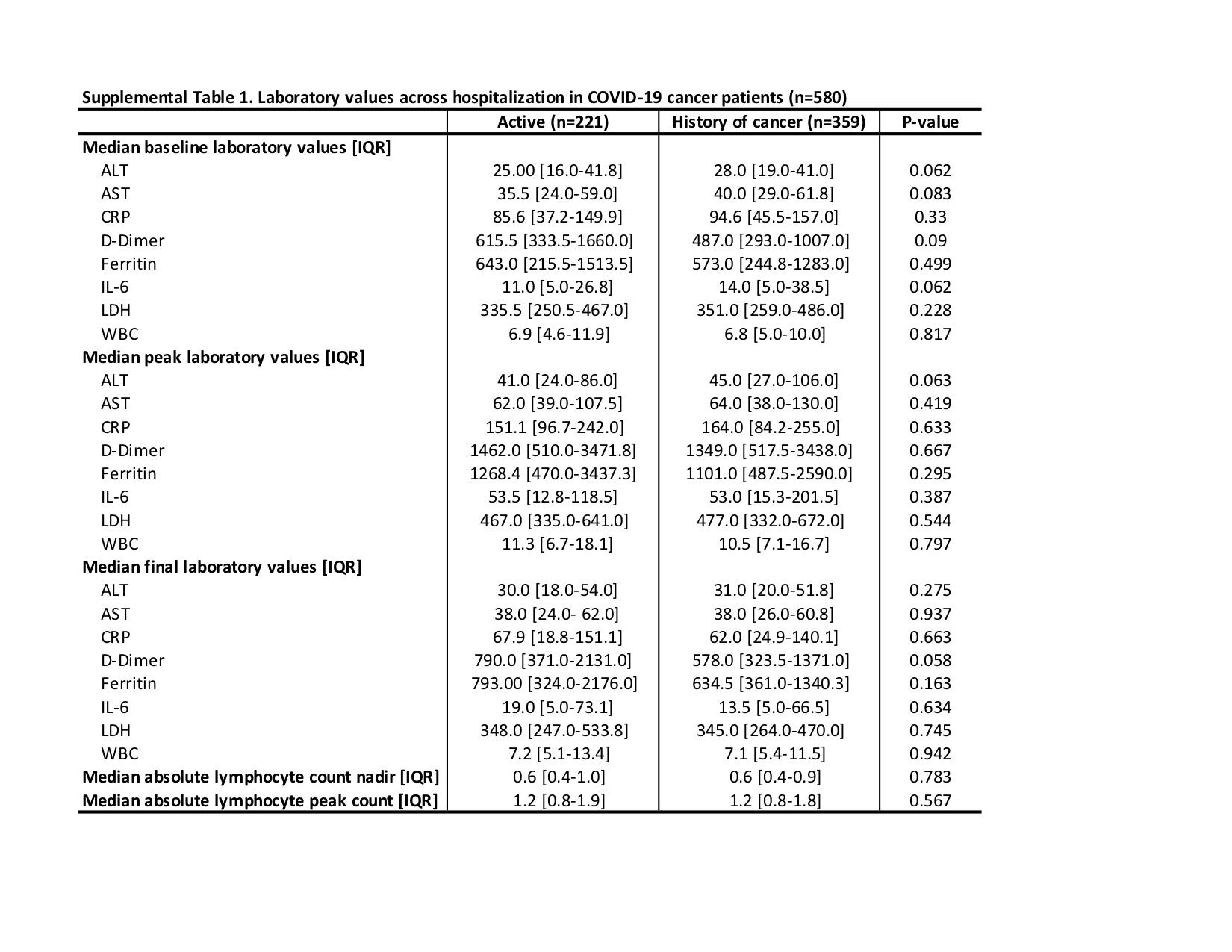

Supplement: Supplementary file 1 — Table S1. Laboratory values across hospitalization in COVID‐19 cancer patients (n = 580). [file CNR2-4-e1413-s002.jpg]

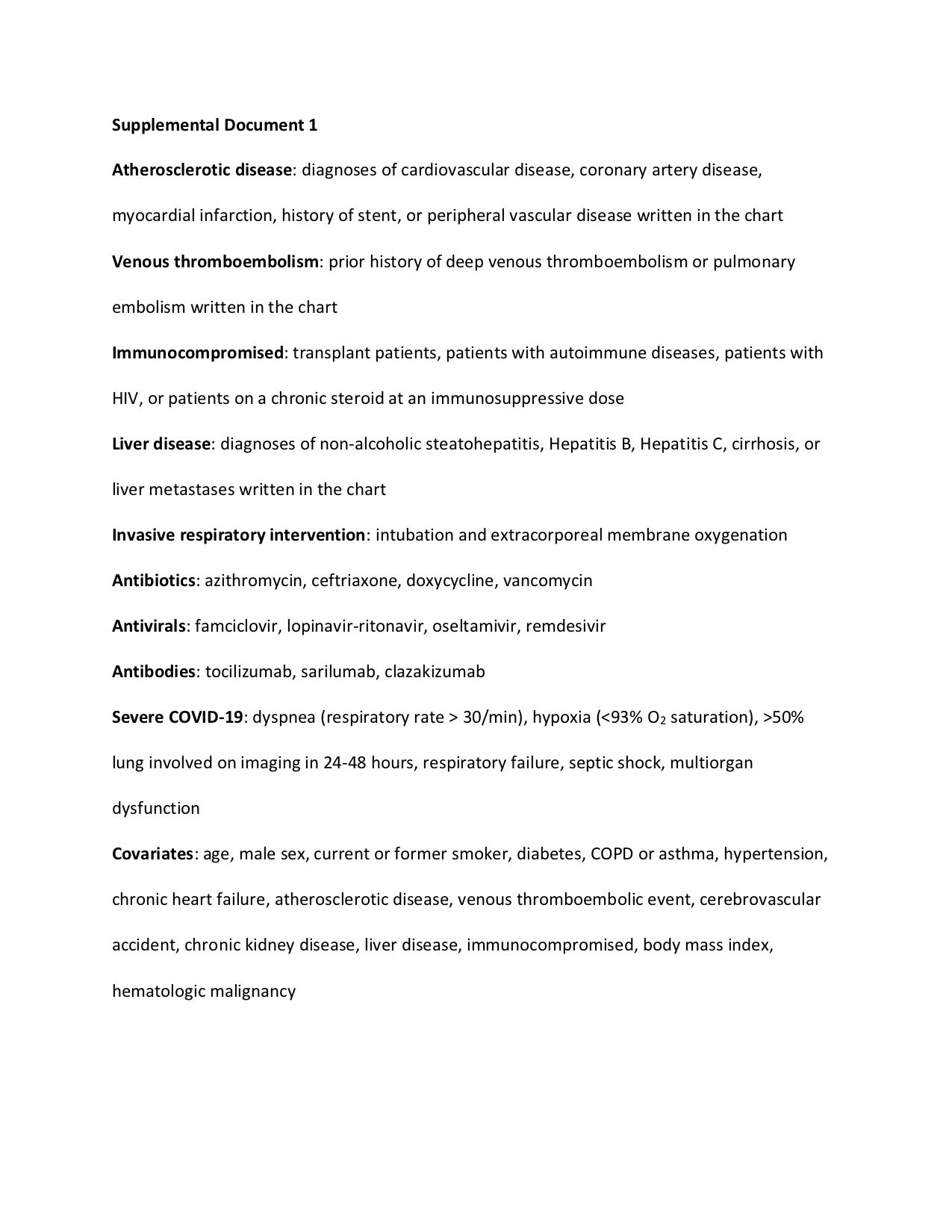

Supplement: Supplementary file 2 — Appendix S1. Supporting information. [file CNR2-4-e1413-s001.jpg]
